# Semi-Annual Meeting of the Veterinary Medical Association of New Jersey

**Published:** 1901-09

**Authors:** George W. Pope

**Affiliations:** Secretary


### SOCIETY PROCEEDINGS.

### SEMI-ANNUAL MEETING OF THE VETERINARY MEDICAL ASSOCIATION OF NEW JERSEY.

THE semi-annual meeting of this Association was held at Stetter's Assembly Hall, 844 Broad Street, Newark, on Thursday, July 11th.

Preceding the regular meeting a clinic was held at the hospital of Dr. Werner Runge, 130 Union Street, Newark. The clinic was under the management of Drs. Runge, McDonough, Hogan, and Hopper. Much credit is due to the members of this committee for the excellent clinic planned and executed by them. Although the attendance was large, the spacious quarters furnished by courtesy of Dr. Runge proved ample for all requirements.

Dr. Runge presented two cases—one of osteoporosis, an aggravated case upon which he had experimented with good results, and another of cancerous growth of a horse's head. Dr. McDonough gave a practical demonstration of the use of stocks. Dr. Miller, the experienced and expert ridgling castrator, performed the operation of castration with the subject in a standing position. Dr. McCully, of New York, with the assistance of Dr. Hopper, performed caudal tenotomy upon several subjects. Dr. McCully is an expert in this line of surgery, and the transformation which he was able to make upon several difficult cases was remarkable and a revelation to the attending surgeons. The Committee on Clinic for the Atlantic City meeting is fortunate in securing Dr. McCully for an operation.

The regular meeting was called to order at 12.30 P.M. by the President, Dr. William Herbert Lowe. Thirty-five members responded to roll-call, and two members were proposed and elected to membership.

Dr. T. E. Smith, of Jersey City, rising on a point of privilege, presented to Dr. Lowe, on behalf of the Association, a handsome gavel, neatly and appropriately inscribed. Although completely taken by surprise, the President replied to the courtesy of his fellow-members in a fitting manner.

It was voted that the Committee on Legislation be authorized to take legal action against those in the State who have registered illegally or are practising without regard to the law, and it is probable that the act to protect the title of veterinary surgeons and to regulate the practice of veterinary medicine and surgery in New Jersey, approved March 4, 1889, will be thoroughly tested in the near future. It was voted that a committee be appointed to draft resolutions expressing the satisfaction of the Association for the noble efforts made by the workers for the Army Legislation bill.

Voted that the Chair appoint a press committee.

The report of the Animal Industry Committee was given by the Chairman, Dr. J. Payne Lowe.

The report of the Committee on Legislation was read by the Chairman, Dr. Budd.

The report of the Public Health Committee was given by Dr. Runge, Chairman.

Drs. Magill and William Herbert Lowe, delegates to the meeting of the Pennsylvania State Association, made their report.

The Treasurer's report showed a balance in the treasury.

A paper was read by Dr. Ancker entitled "Argentum and its Preparations; their Value in Veterinary Practice;" also a paper by Dr. Pope on "Insects in their Relation to the Practice of Veterinary Medicine and Surgery."

The Committee on Arrangements for the Atlantic City meeting reported the need of more funds, and it was voted that a committee of one from each county of the State be appointed to solicit and collect from veterinarians. The Committee of Arrangements, consisting of Dr. A. T. Sellers, Chairman; Drs. J. O. George, E. L. Loblein, J. Payne Lowe, and William B. E. Miller, was accordingly augmented by the following: Dr. T. Earle Budd, for Essex County; Dr. T. E. Smith, for Hudson County; Dr. J. Payne Lowe, for Passaic County; Dr. G. F. Parker, for Mercer County; Dr. Charles E. Magill, for Camden County; Dr. J. B. Hopper, for Bergen County; Dr. James M. Mecray, for Burlington County; Dr. E. L. Loblein, for Middlesex County; Dr. William Gall, for Monmouth County; Dr. James Mosedale, for Morris County; Dr. E. R. Voorhees, for Somerset County; Dr. Whitfield Gray, for Sussex County; Dr. F. A. Zucker, for Union County; Dr. J. M. Everitt, for Warren County.

It was voted that the next regular meeting be held at Trenton; exact place of meeting to be announced later.

George W. Pope,
Secretary.

